# Medium Moderates the Message. How Users Adjust Their Communication Trajectories to Different Media in Collaborative Task Solving

**DOI:** 10.1371/journal.pone.0157827

**Published:** 2016-06-23

**Authors:** Karolina Lisiecka, Agnieszka Rychwalska, Katarzyna Samson, Klara Łucznik, Michał Ziembowicz, Agnieszka Szóstek, Andrzej Nowak

**Affiliations:** 1 The Robert B. Zajonc Institute for Social Studies, University of Warsaw, Warsaw, Poland; 2 University of Social Sciences and Humanities, Faculty in Wrocław, Wrocław, Poland; 3 Cognition Institute, Plymouth University, Plymouth, United Kingdom; 4 University of Social Sciences and Humanities, Faculty in Warsaw, Warsaw, Poland; 5 Design Department, Warsaw Academy of Fine Arts, Warsaw, Poland; University of Rijeka, CROATIA

## Abstract

Rapid development of information and communications technologies (ICT) has triggered profound changes in how people manage their social contacts in both informal and professional contexts. ICT mediated communication may seem limited in possibilities compared to face-to-face encounters, but research shows that puzzlingly often it can be just as effective and satisfactory. We posit that ICT users employ specific communication strategies adapted to particular communication channels, which results in a comparable effectiveness of communication. In order to maintain a satisfactory level of conversational intelligibility they calibrate the content of their messages to a given medium’s richness and adjust the whole conversation trajectory so that every stage of the communication process runs fluently. In the current study, we compared complex task solving trajectories in chat, mobile phone and face-to-face dyadic conversations. Media conditions did not influence the quality of decision outcomes or users’ perceptions of the interaction, but they had impact on the amount of time devoted to each of the identified phases of decision development. In face-to-face contacts the evaluation stage of the discussion dominated the conversation; in the texting condition the orientation-evaluation-control phases were evenly distributed; and the phone condition provided a midpoint between these two extremes. The results show that contemporary ICT users adjust their communication behavior to the limitations and opportunities of various media through the regulation of attention directed to each stage of the discussion so that as a whole the communication process remains effective.

## Introduction

The life of a modern human gradually becomes virtual. Many of us spend more time online than offline. All basic activities including work, shopping, as well as social interactions are effectively transacted in cyberspace. Smartphones, tablets and other electronic devices have gradually and imperceptibly turned into subsidiary ‘limbs’ of our avatar ‘bodies’, used to act and interact in the virtual second life. If this sounds like an exaggeration recall the phantom vibration syndrome (false sensation of phone ringing or vibrating, c.f. [1,2]), similar to phantom pain often experienced after a limb loss. ICT-based communication not only has penetrated the life of individuals, but it has also become the daily bread in modern organizations. It is already a truism to say that companies increasingly rely on technologically mediated interaction to support the work of their teams [3–5]. Working teams use text, voice or video channels or a mixture of those to cooperate effectively regardless of physical distance between communication partners. Face-to-face interaction is no longer the only standard form of social gatherings, what prompts collaborators to accustom to novel conditions of coexistence and joint action [6–9]. Such significant shifts in the technological backing of social interaction impose a daunting challenge on media researchers: to determine how communication practices have changed with the ubiquity of ICT-based media.

One of the most common assumptions is that the effectiveness of communication depends on the richness of the medium. The prominent media richness theory [10] proposes that communication through text, voice or video channels is impoverished compared to the prototypical face-to-face encounter because these media are able to convey less rich information in the same unit of time (so called *cues-filtered models* [11]). Yet, empirical studies comparing the efficiency of task solving through mediated and face-to-face communication give contradictory conclusions [12–16]. Some show that the performance or satisfaction of distributed teams is worse than those interacting face-to-face [4,13,17–20], some show no vivid asymmetries [3,21–27] or even superior performance of distributed teams [28–30]. The equivocality of empirical results led to the development of theories that emphasize the ability to adapt to communication-related obstacles, as team members gain experience with using the medium [30–32]. While still classifying media as less or more efficient or effortful than others [3,33,34], these theoretical propositions call attention to users’ adaptation to media requirements. However, they do not state in what way does the communication change in order to take advantage of and bypass the limitations of a particular medium.

In this paper, we argue that the specific pros and cons of various social media are effectively utilized to reach communication goals through the adoption of a fitting communication strategy (i.e. appropriate time structure and content of conversation). In effect, lean bandwidth is no longer perceived a hindrance for effective communication. Applying social media on daily basis to diverse communication purposes, users choose from a range of possible strategies and apply novel, effective communication routines (conversation trajectories) that work in a context provided by a given medium.

We can hypothesize that this phenomenon, at least in part, is an effect of adaptation. Since ICT has already become the staple of social interaction, we can assume that at least for the younger age groups lack of experience is not an obstacle anymore [35]. Younger users have been brought up with ICT as one of the main communication modes and therefore they are constantly accumulating ICT expertise through the use of miscellaneous communication channels (instant messaging, SMS, phone calls, social media such as Facebook or Twitter). The acquired skills in media usage help communicators take advantage of the pros and cope with the cons of a specific communication channel. A nice example of simultaneous development of experience and technology are text-based media, usability of which used to be seriously handicapped by low typing speed. Nowadays, the necessity to type messages is not as problematic as it used to be due to averagely higher typing skills of media users and technological advancements such as T9 (predictive text technology).

Most media theories conjecture that lean media are unable to support a similar level of message richness compared to face-to-face encounters [36]. Yet, in the majority of empirical studies this claim is validated by contrasting objective or self-evaluated outcome of communication, not its content or dynamics [29]. Only a small fraction of this research examines actual differences in communication patterns, but often only at the level of a single message or the frequency of a particular communicative behavior (e.g. [18,24,29,30]). Even smaller number of studies trace global patterns of group problem solving (e.g. [27,31]). Restriction of media research to differential effectiveness of mediated interaction does not allow for a proper study of users' tactics when they are faced with the specifics of a given communication channel.

We propose that one of the main strategies that help to balance out the leanness of a medium is adjusting the attention devoted to specific parts of the communication process—those phases of conversation that could be hindered by specific properties of a medium are given special attention (reflected in the time development of the conversation) in order to increase the likelihood of mutual understanding. These strategies involve not only grounding efforts [37] at the level of a single communicative exchange, but also more global tactics applied to the whole conversation trajectory. For example, interlocutors may anticipate what kind of information can be easily or poorly transmitted through a given channel and pay special attention to these phases of group or decision development that may be hindered by the lean medium bandwidth. What is more, users are able to take advantage of additional features of a medium and use them to abbreviate or simplify some of the communication stages (especially, if the medium is particularly convenient for this specific phase). One example of such a feature in instant messaging is so called reprocessability of the message [38] which assures that information once contributed is forever accessible for communication partners. This property may significantly facilitate information sharing process.

In order to identify users’ communication strategies in different media, researchers need to look more into the changing content of messages in the communication process and study how teams and individuals manage semantic processing throughout the whole interaction period in different media conditions. A progress is needed from simplistic input-output paradigms towards more process-focused approaches that capture behavioral adjustments of users faced with opportunities and limitations imposed by media. The study reported here was designed to fit that purpose.

## Research Strategy and Hypotheses

In order to investigate how users adjust the communication process in response to conditions imposed by a given medium, we observed dyads interacting to solve a complex task. In organizational setting teams often encounter problems that involve multistage information processing. In complex tasks such as consensus building or decision negotiation team members need to engage in sequentially related stages of joint action in order to come up with an optimal solution. According to the classic division by Bales and Strodtbeck [39], a prototypical sequence of collective problem solving consists of orientation, evaluation and control stages. In the orientation stage relevant information distributed among team members needs to be shared so that the collaborators may build a common knowledge repository. Secondly, the available information needs to be properly assessed with respect to decision criteria and users’ preferences (evaluation stage). Finally, the team must select one decision option that would be satisfactory for all members of the group (control stage).

In the study reported here we compared collective problem solving sequence in 3 media conditions: chat, voice (mobile phone) and face-to-face communication. We expected that using different media for the same task would result in different amounts of time devoted to those stages of problem solving that might be facilitated or perturbed by given media conditions. We also expected that due to high familiarity of modern users with communication technology (especially in the age group of the participants—19–27 years old), performance and satisfaction of distributed teams would be the same as in face-to-face communication.

We have used the “Lost at Sea” problem [40] which is a typical consensus-seeking task. It consists of ordering a list of 15 objects according to their usefulness for survival on a rescue raft (the ordering is first performed individually and later in group setting). “Lost at Sea” problem is an example of a class of negotiation tasks called “survival games” used typically at team building trainings and in small group research [41]. The exercise encourages interaction and teamwork among group members. Participants have to work cooperatively and negotiate the best order of items that can help the team to survive in the open sea. Apart from having a clear criterion of decision correctness, survival tasks tend to evoke rich social dynamics and provide a good indicator of group decision making process [42]. The task was chosen for the present study because it was suited to its main goal, i.e. tracking patterns of social interaction in collaborative task solving.

The complexity of the task lies in the difficulty to align different point of views. The task is usually solved by lay people that lack expertise concerning seafaring. Group members have to exchange and assess the relevance of arguments in support of each item. They are faced with the situation of a high uncertainty, in which the decision options are clear but there is lack of credible information about decision criteria. Often, group process and leadership skills of the particular constellation of group members play a greater role in establishing a decision than the substance of argumentation. Sometimes, even when they are convinced that their opinion is correct, more knowledgeable group members have to sacrifice it for the purpose of establishing group consensus. Arousing both conflict and integration between group members, the “Lost at Sea” task is an adequate tool for observation of decision making processes.

The optimal sequence of actions needed to be undertaken by the team in order to efficiently solve the “Lost at Sea” task clearly follows the sequence proposed by Bales and Strodtbeck [39]. At first, team members need to get information about personal rankings across to the communication partner in order to attain collective orientation in the available decision options. Without proper assessment of the knowledge they possess as a group, coworkers will not be aware of the existing alternatives that need to be considered.

In the middle part of the discussion the challenge is to effectively integrate the knowledge distributed between interacting partners—the evaluation stage. During this stage participants exchange their personal experiences and evaluations to determine the relative importance of listed objects for survival in the open sea. This stage is critical for the task as it leads to collaborators formulating their subjective preferences and integrating distributed information. In contrast, orientation and control may be considered supplementary stages of the decision formation: in the first one users specify optional solutions to be deliberated upon, while the latter is an aftermath of the assessments accumulated in the evaluation stage.

Finally, the final ranking needs to be formed with the approval of all the members of the team. Out of different, often equivocal alternatives the team has to pick one, possibly the most advantageous for the group as a whole. The control stage is thus negotiation of the validity of preferences exchanged during the evaluation stage.

We presumed that transferring complex information in a form that would enable instant comprehension and memorizing was going to be most difficult in the telephone conversation. In face-to-face condition people can simply show their rankings to the partner; in chat, one conversational turn is sufficient to convey the full ranking (which is going to be visible for the partner from thereafter). Phone communication requires partners to carefully listen to each other, to memorize or even to write down their rankings in order to keep them in common knowledge repository. We thus predicted that orientation stage would be most challenging for dyads communicating through audio channel only. This assumption is supported by results of previous studies that compared audio and face-to-face conversations. In audio only communication more instances of grounding are typically observed, as well as more signals of agreement, attentive listening and understanding, and more attempts to elicit feedback from the conversation partner [43,44]—which suggests that transfer of complex information is more difficult through this medium.

For the critical evaluation stage we expected it to be inflated in the condition of face-to-face communication compared to the other two stages. In orientation, visual cues and co-presence in a face-to-face contact enable fast coordination and confident action. Collaborators are able to see each other’s evaluations on the table, they can point to instruction excerpts and objects from the survival list. Therefore, face-to-face teams would presumably spend less time on mutual orientation and control stages and more time on gathering interpretations and on structuring available information. Another reason for which the evaluation stage might be extended in face-to-face condition is its wider social bandwidth (c.f. [45]). As shown in previous research, face-to-face groups tend to spend more time on establishing social relations such as negotiating status, defining task roles and norms of behavior during social interaction [46]. Because of the necessity to exchange personal views and evaluations, the evaluation stage is the most social stage in the Lost at Sea task. Sharing personal opinions requires openness in demonstrating private views and showing interest in the opinion of the other, i.e. it necessitates the emergence of a social relation. For that reason, dyads communicating face-to-face would spend more time in the evaluation stage.

Thirdly, according to media synchronicity theory [38] channels that are lower in synchronicity, such as text-based media, are well suited to information sharing but tend to hinder information synthesis. Synchronous media on the other hand enable fast exchange of short messages between partners, what helps in fluent convergence on a common solution [47]. Based on that, we expected that having no problems with exchanging information during the orientation and evaluation stage, dyads cooperating through chat might experience less ease with effortless validation of arguments and formulating the final decision in the control stage. This fact would be reflected in devoting more time for this stage in text communication than in other conditions.

Last but not least, we expected that due to the compensatory efforts undertaken, performance and satisfaction of the communicators would not be affected by communication mode despite the different richness of the various media.

## Materials and Methods

Thirty-five, same gender dyads were observed while solving the “Lost at Sea” problem [40] communicating via 3 media: chat (11 dyads), face-to-face (12 dyads), mobile phone (12 dyads). The participants were instructed to reach a consensus concerning the ordering of the provided list of objects according to their usefulness for survival on a rescue raft. Before starting the dyadic discussion partners solved the task individually. The conversations were recorded or videotaped as appropriate and then transcribed. Afterwards, each conversational turn was assessed by independent judges as either belonging to orientation, evaluation or control stage of the problem solving process. Additional measures (performance, satisfaction, interaction attitudes as well as perceived media richness) were gathered after the discussion.

### Ethics Statement

All procedures were approved by the Research Ethics Board at the Robert B. Zajonc Institute for Social Studies, University of Warsaw. Potential participants in the study were informed about its general aim, duration, outline of the procedure and remuneration. They were also told that participation is voluntary, that they can withdraw from the study at any point without telling the reason, and that all data is anonymous and will only be used for reasons related to this study. All participants confirmed having understood the received information and gave their oral consent prior to participation.

### Participants

Participants in this study were 70 (49% female) inhabitants of student housing facilities in Warsaw, aged 19–27 (*M* = 21.43, *SD* = 1.73). They were enrolled to participate in the study by external recruiters and received remuneration for participating in the study (the equivalent of 15 euros in local currency).

### Procedure

When participants arrived at the lab, they were paired into same-sex dyads and randomly assigned to one of three experimental conditions: face-to-face, mobile phone or Internet chat communication channel. First each participant went to a separate computer laboratory and completed the experimental task individually. Then participants completed the same experimental task together with their partner using the assigned communication channel (in mobile phone or internet chat communication conditions participants stayed in their laboratories; in the face-to-face condition one of each dyad went to their partner’s laboratory to complete this part of the study and then returned to their laboratory). Finally, each participant individually filled out interaction attitudes, media richness, negotiation process and its outcome satisfaction questionnaires. When the study was over participants were thanked, carefully debriefed and given their remuneration. The procedure took about 30 minutes. The anonymized data files used for the analyses are available from the Figshare database (accession link http://dx.doi.org/10.6084/m9.figshare.1452891).

### Experimental task—Lost at Sea

In the individual variant of the procedure, the participant is asked to imagine that s/he is a castaway on a sinking yacht, hundreds of miles away from the nearest landfall. S/he managed to salvage a rubber life craft and 15 items (a sextant, a shaving mirror, a quantity of mosquito netting, a 25 liter container of water, a case of army rations, maps of the Atlantic ocean, a floating seat cushion, a 10 liter can of oil/petrol mixture, a small transistor radio, 20 square feet of opaque plastic sheeting, a can of shark repellent, a bottle of rum, 15ft nylon rope, 2 boxes of chocolate bars, a fishing kit), but the raft is too small to fit all of them. The task is to pick five most appropriate items and rank them from the most important for survival, in their opinion, to the least important one.

In the dyadic variant of the procedure, participants are asked to imagine that they are castaways on a sinking yacht together. They managed to salvage a rubber life craft and the same 15 items. Their task is to agree on five items that are the most important for survival and come up with a common ranking of the items from the most important to the least important one. While picking and ordering the items they have to communicate using the assigned communication channel depending on the experimental condition (face-to-face, mobile phone or internet chat). There was no time constraint on either the individual task or on the dyadic conversation.

In the usual procedure, the task is first solved individually and then cooperatively. Most of the times, team score is higher than the average scores of team members. This confirms the assumption of the “wisdom of the crowd” effect [48]: when people from different backgrounds meet to make a decision, each of them has a different tool set (i.e. knowledge and experience) used to validate the best choice. Team’s tool set is then the sum of the knowledge and experience of the members. Therefore, the more individual tool sets there are within a team and the more diverse they are, the more accurate the final group decision [49].

Lost at Sea exercise has a single best solution, provided the US Coastguard—the five items most important for survival are: (a) a shaving mirror; (b) oil/petrol mixture; (c) water; (d) army rations; and (e) plastic sheeting.

### Measures

#### Questionnaires

**Interaction attitudes** were measured using an abridged version of the Affective Benefits and Costs in Communication Questionnaire [50]. The scale consists of six subscales: personal effort, sharing experiences, achieving recognition, group attraction, invasion of privacy and processing effort. Altogether the scale has 18 statements, agreement with which is measured on a 7-point Likert-type scale. Sample questions from each subscale are: *I put effort into making the contact nice for the other* (personal effort); *I found it difficult to share experiences with the other through this medium* (sharing experiences); *I know what the other feels during a contact* (achieving recognition); *I feel a bond with the other because of the contact* (group attraction); *Through our contacts*, *the other learns more about me than I would like him/her to know* (invasion of privacy); *I could have made some more effort during our interaction* (processing effort).

**Perceived media richness** was measured using Media Richness Questionnaire [51]. The scale consists of eight statements, agreement with which is measured on a 7-point Likert-type scale. Sample questions are: *When we disagreed*, *the communication conditions made it more difficult for us to come to an agreement; The conditions under which we communicated slowed down our communications*.

**Negotiation process satisfaction** was measured using Process Satisfaction Questionnaire [51], which has three subscales: process satisfaction, other’s influence and my influence. The questionnaire consists of six questions answered on a 7-point Likert-type scale. Sample questions are: *To what extent did the opinions of the other person make you change your mind*? *How much did you hold on to your opinions*?

**Satisfaction with the outcome of the negotiation** was measured using Outcome Satisfaction Questionnaire [51]. The questionnaire consists of six questions answered on a 7-point Likert-type scale. Sample questions are: *How satisfied or dissatisfied are you with the quality of the solution*, *which you and the other party reached*? *To what extent are you confident that the solution is optimal*?

#### Task measures

**Quality of solution** to the Lost at Sea task was quantified as a similarity between a given solution and the optimal solution described above. To calculate how accurate a ranking was, we granted participants points that were based on the positions of each item in the ranking provided by the Coastguard. For example, if a person indicated that s/he would pick a shaving mirror, s/he would be granted 15 points (maximum) for that decision—because this item is considered the most important by experts. If a person indicated a sextant (least important for survival), s/he would be granted only 1 point. The ordering of the 5 items chosen by the participants was not taken into account when calculating their score. The quality of the final ranking was calculated as the sum of the points gathered by the participant for indicating each of the five items. Thus the maximum score that could be obtained was 65 points (if all 5 items that topped the expert ranking were chosen by participants) and the minimum was 15 points (the 5 lowest ranked items were chosen by the participant).

**Quality of solution for the dyadic choice** was computed in exactly the same manner as for individual solutions.

**Improvement of solutions** was quantified as the difference between the mean quality of individual solutions of partners in the dyad and the quality of their dyadic solution.

**Time to reach solution** was measured (in seconds) only for the dyadic part of the task.

#### Discussion phases

Phases of the dyadic problem solving process were assessed using a competent judges procedure. Each utterance was first independently categorized by two independent judges as belonging to either the orientation, evaluation or control phase [39]. The judges’ agreement at that point was substantial [52], Cohen’s κ = 0.77, p < .001, but to improve it even further they were nevertheless asked to reevaluate their assessments. The two judges discussed together these utterances which they categorized differently, until they have reached an agreement. All analyses were done on the final common categorization agreed on by both competent judges.

To compare the **proportions of discussion phases** between media we have computed for each dyad proportions of utterances that belonged to each phase: orientation, evaluation and control. This resulted in an aggregated dataset with each dyad as observation (N = 35).

To assess the **trajectory of the discussions** we have divided the utterances in each dyads’ discussion into 10 consecutive bins, each containing 10% of the total number of utterances for the given dyad. We have then computed the proportions of discussion phases in each bin, similarly to the computation for the whole discussion.

To measure the **transition probabilities** between the phases we have categorized each consecutive pair of utterances of each dyad as belonging to one of the 9 possible transitions between the three phases (orientation-orientation, orientation-evaluation, orientation-control, evaluation-orientation, etc.). For each dyad we have thus obtained a series of categorized transitions of which length was equal to the length of the conversation less one (the last utterance was not followed by any other). Then for each phase in each dyad we have computed the proportion of transitions that led to each phase in the next utterance.

## Results

### Descriptive statistics

We first compared the descriptive statistics of all individual level variables in three experimental conditions (see Table 1). The only observed differences between them were in media richness and sharing experiences. Dunnett’s C post-hoc test revealed that chat was perceived less rich a communication medium than phone and face-to-face contact. Bonferroni’s post-hoc test revealed that the sharing experience was perceived as worse in chat than in phone communication, with face-to-face not significantly different from either. Other than that, the three communication conditions were rated equal in terms of interaction attitudes, as well as satisfaction from the negotiation process and its outcome. There were also no differences between experimental conditions in age and gender distribution of participants.

**Table 1:** Descriptive statistics of individual level variables used in the study.

Variable	Chat	Phone	Face-to-face	Group comparison
Age	*M* = 21.32, *SD* = 1.76	*M* = 21.62, *SD* = 1.86	*M* = 21.33, *SD* = 1.63	*F*(2,67) = 0.23, *p* = .79
Gender	55%F	42%F	50%F	**χ** ^2^(2) = 0.79, *p* = .67
Quality of individual solution	*M* = 44.09, *SD* = 8.62	*M* = 46.63, *SD* = 7.44	*M* = 45.25, *SD* = 5.95	*F*(2,67) = 0.68, *p* = .51
Personal effort	*M* = 5.41, *SD* = 1.35	*M* = 5.65, *SD* = 1.25	*M* = 5.63, *SD* = 1.16	*F*(2,67) = 0.26, *p* = .78
Sharing experiences	*M* = 3.59, *SD* = 0.80	*M* = 4.49, *SD* = 0.92	*M* = 4.11, *SD* = 1.02	*F*(2,67) = 5.50, *p* = .01
Achieving recognition	*M* = 4.41, *SD* = 1.33	*M* = 4.99, *SD* = 1.13	*M* = 4.97, *SD* = 0.93	*F*(2,67) = 1.91, *p* = .16
Group attraction	*M* = 4.58, *SD* = 1.32	*M* = 5.08, *SD* = 1.22	*M* = 4.93, *SD* = 1.18	*F*(2,67) = 1.01, *p* = .37
Invasion of privacy	*M* = 1.70, *SD* = 0.77	M = 1.96, SD = 0.98	M = 1.78, SD = 0.77	*F*(2,67) = 0.58, *p* = .56
Processing effort	*M* = 3.52, *SD* = 0.79	*M* = 4.14, *SD* = 1.07	*M* = 3.99, *SD* = 1.05	*F*(2,67) = 2.50, *p* = .09
Media richness	*M* = 4.22, *SD* = 1.37	*M* = 5.63, *SD* = 1.07	*M* = 6.16, *SD* = 0.76	*F*(2,67) = 19.29, *p* < .001
Negotiation process satisfaction	*M* = 5.92, *SD* = 0.80	*M* = 6.33, *SD* = 0.64	*M* = 6.11, *SD* = 0.78	*F*(2,67) = 1.72, *p* = .19
Other’s influence	*M* = 4.46, *SD* = 1.06	*M* = 4.59, *SD* = 0.52	*M* = 4.66, *SD* = 0.97	*F*(2,67) = 0.29, *p* = .75
My influence	*M* = 4.36, *SD* = 1.36	*M* = 4.42, *SD* = 0.97	*M* = 4.75, *SD* = 1.33	*F*(2,67) = 0.68, *p* = .51
Outcome satisfaction	*M* = 5.43, *SD* = 1.03	*M* = 5.87, *SD* = 0.63	*M* = 5.74, *SD* = 0.82	*F*(2,67) = 1.63, *p* = .20

### Dyadic level

**Fig 1:**
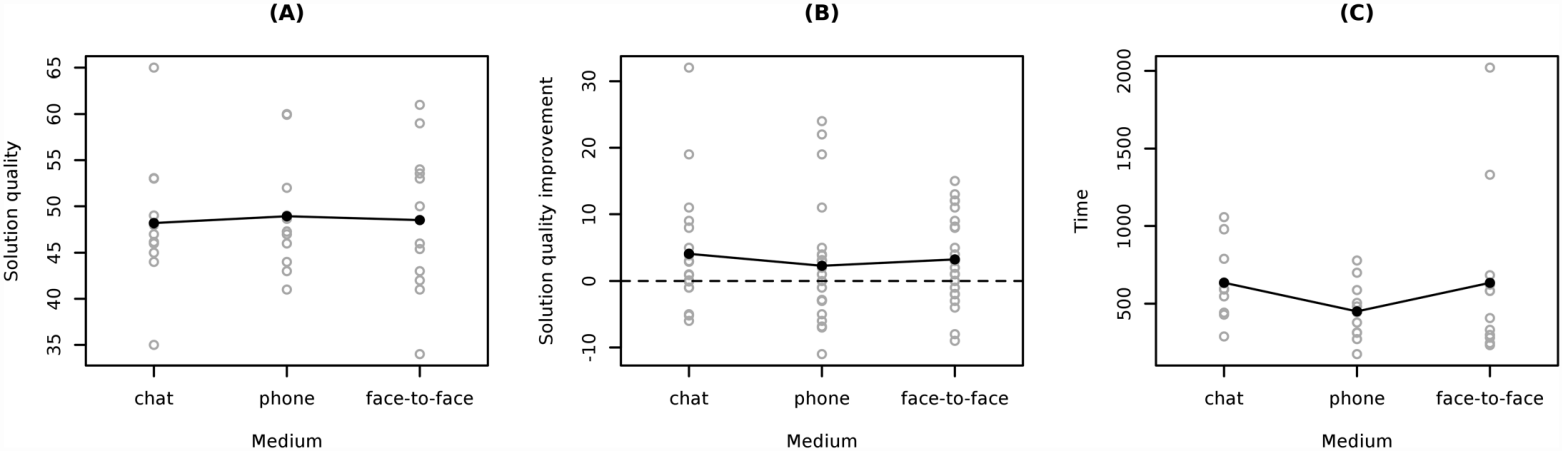
Comparison of the quality of solutions achieved by dyads (A), difference between the quality of dyadic and individual solutions (B), duration of conversations (C) in three conditions: chat, mobile phone and face-to-face communication.

The quality of solution reached by dyads in the Lost at Sea task was the same in all experimental conditions, chat (*M* = 48.18, *SD* = 7.40), phone (*M* = 48.92, *SD* = 5.98) and face-to-face communication (*M* = 48.50, *SD* = 7.93), *F*(2,32) = 0.31, *p* = .97. For all the tested dyads the solution improvement from individual to group score was significant (F(1,32) = 11.41, p < .005)). However, the improvements did not differ across the three experimental conditions, chat (*M* = 4.09, *SD* = 8.39), phone (*M* = 2.29, *SD* = 8.92) and face-to-face communication (*M* = 3.25, *SD* = 6.60), *F*(2,67) = 0.29, *p* = .75. The average amount of time (in seconds) needed to reach the solution also did not differ between experimental conditions, chat (*M* = 635.36, *SD* = 230.75), phone (*M* = 450.17, *SD* = 177.06) and face-to-face communication (*M* = 634.75, *SD* = 531.89), *F*(2,32) = 1.08, *p* = .35. A comparison of the three experimental conditions in terms of time and quality of achieved solution is presented in Fig 1.

**Fig 2:**
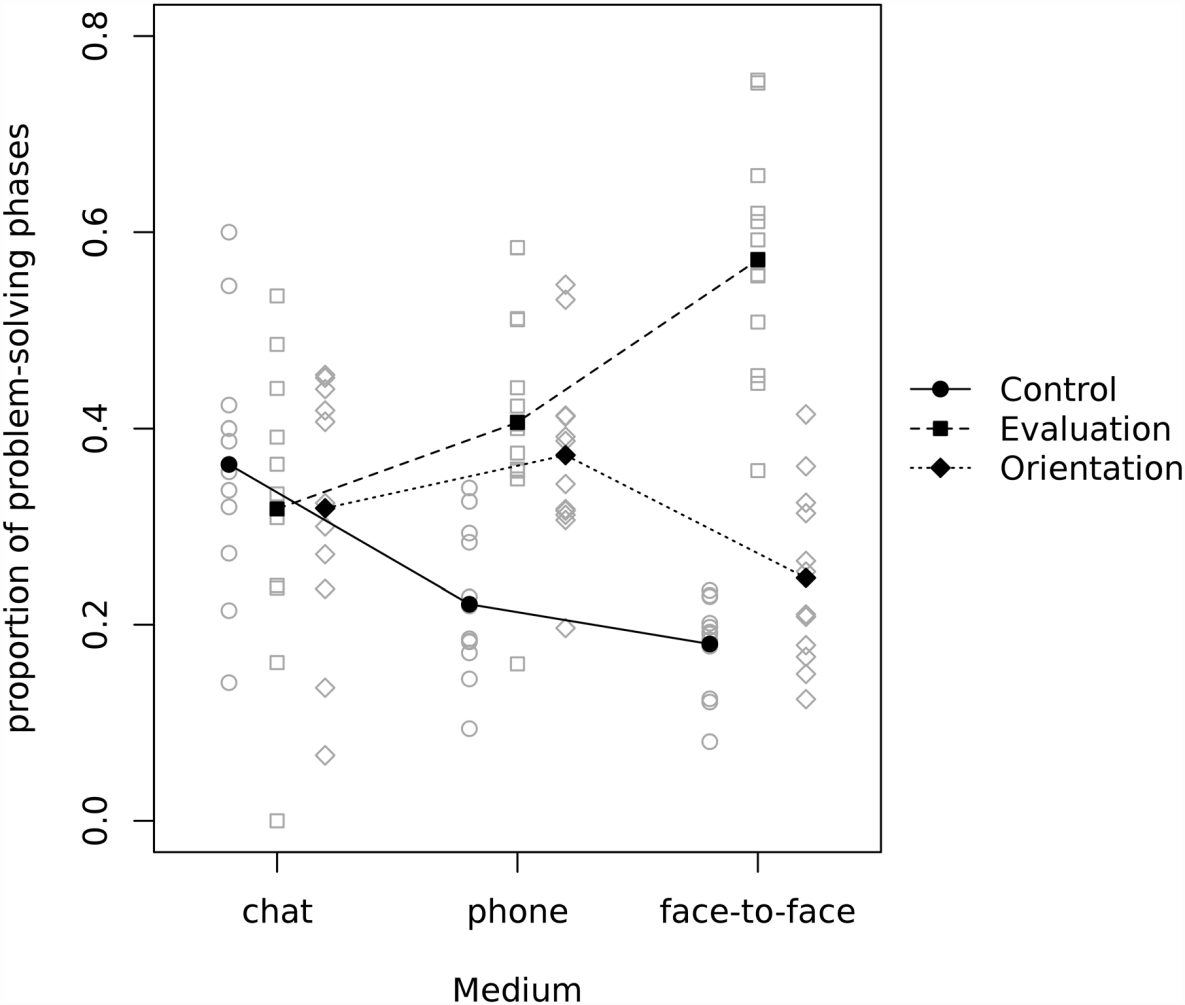
Proportions of utterances devoted to three problem solving phases (orientation, evaluation, control) in chat, mobile phone and face-to-face communication.

There were significant differences in the amount of orientation, evaluation and control while performing the Lost at Sea task through different media, as indicated by the results of one-way analyses of variance with Bonferroni (orientation and evaluation) and Dunnett’s C (control) post-hoc tests. There was more orientation over the phone (*M* = 0.37, *SD* = 0.10) than during face-to-face contact (*M* = 0.25, *SD* = 0.09), with chat (*M* = 0.32, *SD* = 0.13) not significantly different from either of the other two conditions, *F*(2,32) = 4.09, *p* = .03. Evaluation dominated the face-to-face condition (*M* = 0.57, *SD* = 0.12), while phone (*M* = 0. 41, *SD* = 0.11) and chat (*M* = 0.32, *SD* = 0.15) did not differ in this regard, *F*(2,32) = 11.89, *p* < .001. Control was more present in chat communication (*M* = 0.36, *SD* = 0.13) than over the phone (*M* = 0.22, *SD* = 0.08) or face-to-face (*M* = 0.18, *SD* = 0.05), *F*(2,32) = 12.68, *p* < .001. Proportions of each of the problem solving phases in the three experimental conditions are presented in Fig 2.

**Fig 3:**
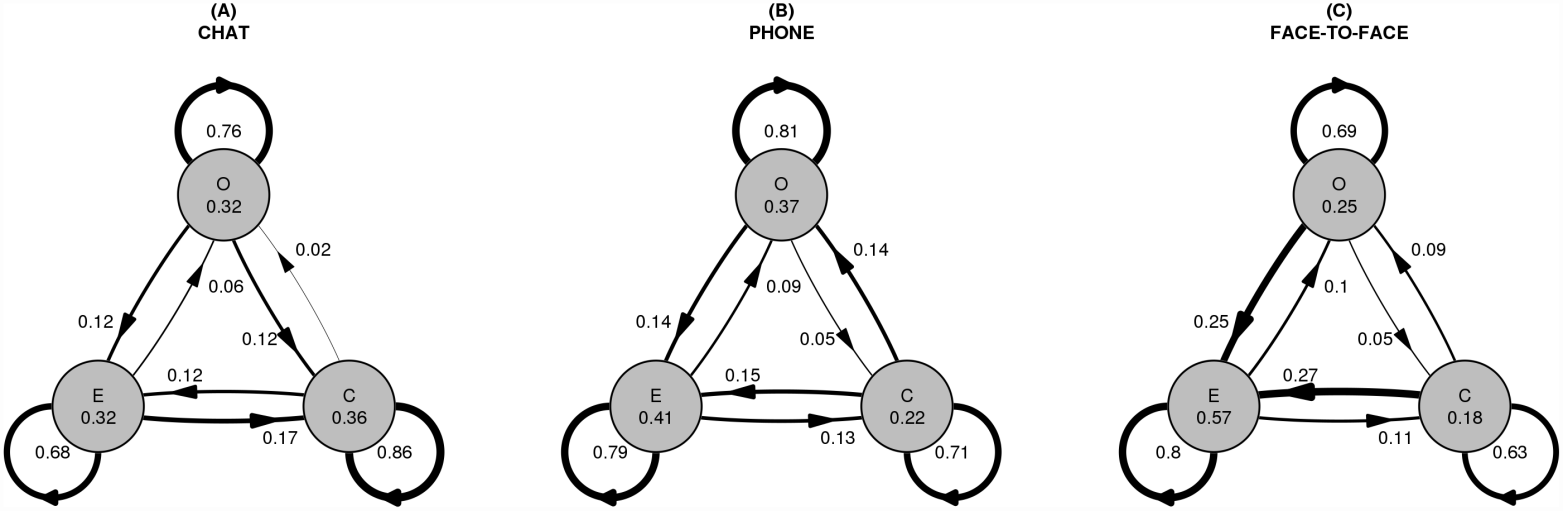
State transition diagrams in 3 media conditions: chat (A), mobile phone (B), face-to-face (C).

We also investigated the sequential structure of dyads’ movement through the stages of task solving. Transitional probabilities were calculated for each dyad to analyze what were the odds of moving from stage to stage. Fig 3. presents state transition diagrams aggregated for each medium. Each arrow represents the mean value of a transitional probability in that medium.

Having the transitional probabilities calculated for each dyad, we could use them as scores in analysis of variance and test the significance of the differences in state transition probabilities between media. The ANOVA analysis with Bonferroni post-hoc tests showed that movement from orientation to evaluation was significantly more probable in face-to-face (*M* = 0.25, *SD* = 0.1) than in chat (*M* = 0.12, *SD* = 0.11) or phone (*M* = 0.14, *SD* = 0.07); *F*(2,32) = 7.48, *p* < 0.005. The same was true for the control to evaluation transition. Here, because the homogeneity of variance assumption was violated (F = 3.45, p = 0.04), Brown-Forsythe test was used, which showed that the transition was significantly more probable in face-to-face (*M* = 0.27, *SD* = 0.13) than in other conditions—phone (*M* = 0.15, *SD* = 0.09) and chat (*M* = 0.12, *SD* = 0.08); *F*(2,28.42) = 6.88, *p* < 0.005).

Moreover, in chat, dyads tended to less rarely move from control to orientation stage (*M* = 0.02, *SD* = 0.04) than in phone (*M* = 0.14, *SD* = 0.09) or face-to-face (*M* = 0.09, *SD* = 0.05); *F*(2,32) = 9.29, *p* = 0.001. Once they reached the control stage, the dyads in chat condition tended to stay in that stage (*M* = 0.86, *SD* = 0.1) more than in other conditions (*M* = 0. 71, *SD* = 0.11 for phone, *M* = 0.63, *SD* = 0.14 for face-to-face condition); *F*(2,32) = 10.15, *p* < 0.001.

The graphs in Fig 3 plainly show that staying in the same stage in the next time step tended to be markedly more probable than moving to another stage. To prove this, we constructed a binomial variable which scored 1 each time a stage was the same in the subsequent turn, and 0 when the stage was different. One sample binomial test was calculated for each dyad, which showed that for only 3 out of 35 dyads moving from one stage to another was equally probable as staying in the same stage.

**Fig 4:**
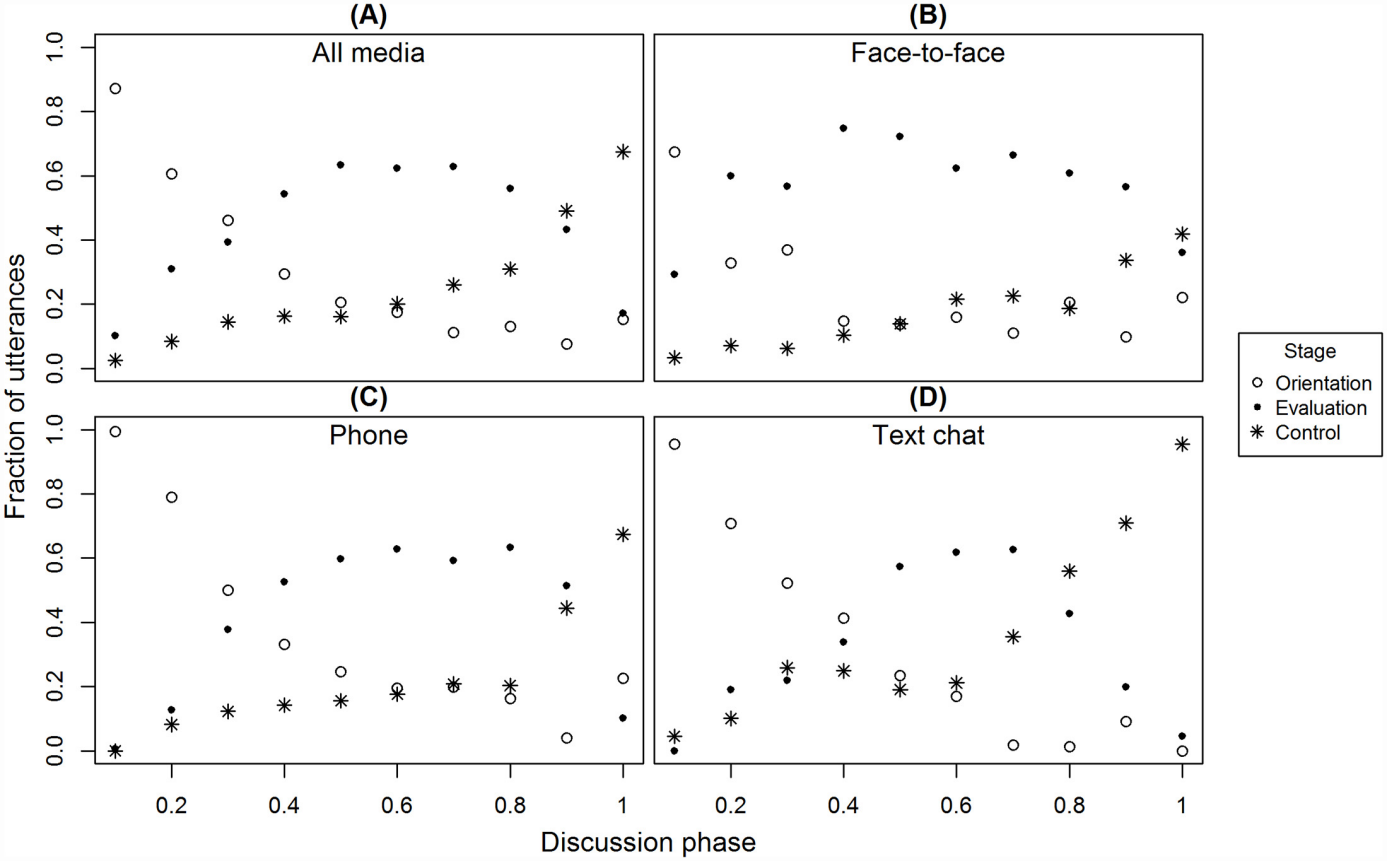
Trajectories of discussion stages in 3 communication conditions (face-to-face, phone and chat) and averaged over all media. On X-axis each point refers to a fraction of the total number of utterances ordered in time. The Y-axis depicts the fraction of utterances of each category in the given time frame.

To fully understand how each phase develops and transforms into another, we have analyzed the trajectory of each discussion phase. Fig 4 shows how the proportions of discussion phases change over the course of the interaction (for all media—A) and for each medium separately—B) through D). In most of the time windows one dominating phase can be indicated. At the level of conversation as a whole we can observe a global pattern of phase transitions from orientation through evaluation with ending control phase. At the beginning team members engage in orientation about the task and its requirements, they present their individual rankings. Once the rankings have been exchanged, the evaluation process overtakes the discussion. After exchanging relevant information, a final group ranking can be decided upon what occurs during the control stage. This sequence was observed in its most pure and orderly form in chat conversations. The phases are most evenly distributed in chat and slightly less so for phone conversations. In face-to-face condition the evaluation phase dominates the discussion, with only a slight prevalence of orientation at the beginning of the interaction and a minimal difference of control at the end.

## Discussion and Conclusions

In the ever-changing technological space, the human communication practices are changing as well. On the one hand, new, sophisticated features are constantly being implemented into ICT tools and platforms to allow for even more intuitive social interaction in the virtual space. On the other, the plasticity of human cognition and behavior enables users to adjust to any situational context in which social interaction takes place and to maintain high levels of communicative performance.

The results of our study support the thesis that current ICT users are able to balance out any pros and cons of the imposed communication channel and they do so through adjusting of communication patterns. The first finding presented here that justifies this conclusion is that communication effectiveness did not differ between media—the quality of solutions to the given task was the same under conditions of face-to-face, phone, or Internet chat interaction. What is more, not only the outcome of communication did not depend on the medium used, but also no differences between text, audio and face-to-face conditions were observed in satisfaction from the actual process, its outcome and other psychological variables, such as perception of own influence or experienced processing effort. This suggests that not only users are able to utilize the pros and cons characterizing a particular channel (even if it is imposed) but that they also perceive the new media as natural—both for successful communication as well as for relation building and socializing. The only differences between text, mobile phone and face-to-face conditions in our results were related to perceived lower media richness and sharing experience in the chat condition. Unequal assessment of communication richness in our study confirms previous scientific findings [14]. It has been shown that even though comparative levels of performance are being attained in leaner media, communication partners still tend to assess them as less rich.

Our results confirm previous findings that show no differences in the effect of medium on the quality of communication outcome, nor the satisfaction of users [3,21–27]. Using the same survival task, Roch and Ayman [53] found no difference in the quality of solutions produced by the teams working face-to-face and via computer chat. Also, Thompson & Coovert [41] found no effects of the medium on the quality of solutions in the “Desert Survival” Task, however the process improvement technique that they used (stepladder technique) work better for the face-to-face than computer-mediated teams. Setlock et al [54] found no differences between communication type (face-to-face or instant messaging) or American and Chinese culture groups. This line of research contradicts predictions of the cue-filtered models, which assume that the lean bandwidth of the new media limits communication. The already discussed media richness theory [10] assumes that high levels of performance can be maintained only when capacity of a medium is well fitted to complexity of a collaborative task. Leaner media are better suited to unequivocal communication, whilst ambiguous, non-routine tasks require the medium of communication to be richer [55].

This assertion constricts research attention to passive reactions of users to limitations imposed by media—users are thought to simply choose the most suitable medium. In order not to face communication failure, they are expected to avoid certain experiences instead of altering their behavior. Our results posit a challenge to such theories as they show that even if a communication channel is imposed on interlocutors (with the task kept the same), the users deal with the media specifics—limitations and opportunities—in such a way as not to hinder the effectiveness of communication.

A possible explanation of such well-fitted communication strategies can be found in the theories that emphasize active adaptation of human behavior in response to communication-related obstacles. One such attempt is *media naturalness theory* [14,30,31], and its more recent incarnation, *media compensation theory* [3,22]. Their authors take up an evolutionist perspective to point out that human brains evolved in conditions of face-to-face communication and thus are not adapted to other forms of communication. However, humans are able to perform compensatory adaptation to new technologies by “substantial behavioral alterations” that balance unnaturalness of media [3]. In the context of relationship building, *social information processing model* [32,56,57] predicts that less personal style of text-based communication can be reduced if partners are given enough time to develop mutual feelings of closeness and trust. Experiential factors are also emphasized in *channel expansion theory* [58], which claims that practical experience with using a communication channel is a necessary requirement for a satisfying mediated social interaction and positive evaluations of medium’s richness. The compensatory effect of time inferred by these theoretical accounts has been confirmed in numerous studies [27,45,44,59,60], being an important indication for involvement of adaptation and learning processes in media use.

Even though the abovementioned theories accentuate users’ accommodation to media requirements, still media other than face-to-face are considered an obstacle rather than an equally effective means of information transfer [3,22,28,44,41,49]. In contrast to this we propose that for contemporary users ICT-based communication has become similarly natural and intuitive as face-to-face contacts. Especially in the younger age group—which is constantly immersed in a technology mediated social world and which is naturally cognitively flexible—the users are able to volitionally compensate for constraints and shortcomings imposed by media [22,33,34]. They do so, as we have shown here, without losing out on effectiveness or performance quality. This active adjustment to the specific communication conditions allows users to apply new media even to sophisticated, multistage, cooperative tasks.

The second finding of our study—which complements the first result of equal effectiveness in various media—explains how users are able to adjust so well. DeLuca et al. [22] admit that “compensatory adaptations are not currently operationalized in the literature” (p. 69). One reason for this is the fact that majority of empirical studies on media compare objective or self-evaluated outcome of communication, not its content or dynamics [29]. Only a small fraction of media research examines actual differences in communication patterns, usually at the level of a single message or communication style (e.g. [18–20,24,29,30,41,50,51]). Even smaller number of studies trace global patterns of group problem solving (e.g. [27,31]). Due to the simplistic input-output approach dominant in the field, the adjustment hypothesis has been so far inferred from equal performance of teams interacting via different media, rather than proved on the basis of extensive observation of communication patterns.

Our results add new evidence to the scarce research on the dynamics of mediated social interaction by proving that the tuning of users’ behavior to a specific medium does not stop at adjusting single utterances or communicative exchanges but affects the whole span and trajectory of an interaction. That is, the time pattern of communication (its content and phases) was also fitted to each medium. We can assume that these patterns were due to specific limitations and benefits offered by different communication channels: if a particular phase is made more difficult by media limitations, the time spent in it should be longer when communicating through that medium than through media that do not have such limitations.

We show that users tend to devote different amounts of time to the problem solving phases proposed by Bales and Strodtbeck [39] when communicating via different media. In text-based asynchronous communication, each phase of problem solving is relatively similarly absorbing, with the final decision making process (control phase) taking relatively more space compared to other media. Written communication apparently simplifies information sharing but hinders its integration into a cohesive conclusion (c.f. [33]); therefore, in a text chat more time needed to be dedicated to overcome this obstacle. In comparison, face-to-face communication was dominated by agreeing on a shared evaluation and on negotiation of relative importance of items in the “Lost at Sea” task. The other two phases—orientation and control—were relatively short in face-to-face condition. Both of these phases require simultaneous focus on transmission and apprehension of the message received from the sender, and direct contact may have facilitated coordination between these two contradictory processes. Moreover, the social character of face-to-face communication, which tends to be focused on status negotiations and establishing group roles and norms [46] and might also promote openness in presenting personal views and evaluations, might have contributed to prolonging the evaluation stage of decision making.

Even though our findings confirm the hypothesized high dedication to orientation phase in the mobile phone condition, the difference from other media in the length of this phase was not pronounced. The significant difference was between face-to-face, where orientation phase was relatively the shortest and mobile phone, where it was the longest. It appears that the main demarcation between media lies in the co-presence feature of face-to-face communication, i.e. "conditions in which human individuals interact with one another face to face from body to body" [61]. Verbal communication without cues provided by co-presence (presumably the ability to point to objects in a common space) appeared to be the most disorienting for users and required additional efforts reflected in the extension of orientation phase in mobile phone condition. Chat condition lacks the co-presence cues as well and it might be the reason why the orientation phase here did not significantly differ in length from the phone condition.

The statistical differences between the media in the amount of each phase in conversations can be explained by a detailed analysis of the trajectories of the discussions. Analysis of transitions between the phases revealed that in all conditions, dyads were significantly more eager to reside in the same stage in the subsequent turn than to change one. This result reflects the fact that conversation is not a random series of unrelated events but rather it tends to be organized into coherent threads in which topics are being deliberated until the conclusion is reached. For face-to-face conversations, the central area of the stage transition diagram was the evaluation phase. Teams working face-to-face tended to return to evaluation from other stages more often than in other conditions. Because evaluation is the most social stage, as noted earlier, and because face is the most effective transmitter of social cues, partners in face-to-face condition might feel tempted to exchange more social statements regarding the discussed issues, such as private views and opinions.

Another finding revealed by the analysis of transitional probabilities concerns the role of the control stage in the chat condition. In chat dyads were significantly less likely to go back from control to orientation and tended to stay in the control stage once it was entered. We suspect that to some extent the sequential structure of decision development is reproduced at the level of a single discussion thread, especially for the media with higher bandwidth. For example, in the case of the sextant (one of the items in the “Lost at Sea task”), team members might first try to orient themselves in what this item is. Then, after assessing its usefulness through an exchange of evaluations, they might decide if they are willing to take it on the rescue raft and how crucial it is for survival. Once the item has been considered, the team may move on to discussing a next one. In chat condition, such a process would be more difficult due to the relatively high cost of coordination when switching between such cycles of assessment. Therefore, when texting, the partners might be more inclined to discuss the whole list at once and thus complete the discussion cycle only once.

These results are further clarified by analyzing the time course of each discussion stage. Diagrams of phase trajectories show how each phase was distributed throughout the discussion. In most of the time windows one dominating phase can be indicated. At the level of the conversation as a whole we can observe a global pattern of stage transitions from orientation through evaluation and ending with the control phase. At the beginning team members engage in orientation about the task and its requirements, they present their individual rankings. Once the rankings have been exchanged, the evaluation process overtakes the discussion. After exchanging relevant information, a final group ranking can be decided upon, what occurs during the control stage.

However, the diagrams also indicate that there are no clear-cut transitions between phases. They show that each discussion stage is a dynamic process and not an all-or-nothing phenomenon. Each phase has a unique pattern of time development. Orientation is high at the beginning and then continuously decays over the course of the discussion, evaluation grows, reaches its maximum and then diminishes, and control starts low but grows in importance as the talk moves on.

What is important, however, is that in any given period there is high chance that utterances of all types will appear. This indicates that the activities represented by each phase tend to be pursued in parallel. The differences along the discussion timeline are not in the presence or absence of any stage but rather lie in the different proportions of utterances of each phase. In the beginning, orientation dominates, but there are already seeds of both evaluation and control phases. As the discussion flows on, orientation subsides and evaluation takes over with control slowly gaining more attention. Finally, evaluation recedes and control takes the lead.

While this general pattern is clearly visible if we average phase proportions over all media (i.e. it represents the most general tendency) and can be traced in the discussions over each medium separately, it is also clear that media differ in the way the phases play out throughout the discussion. For text chat, the domination of the phases is almost evenly split—in the first 33% of the discussion orientation is most visible, in the next 33% evaluation is dominant and control tops the last 33%. In conversations over the phone the first 33% of the discussion are similarly occupied mostly by orientation. Further the line however, the balance changes compared to text chats. Once evaluation takes over, it dominates the next 50–55% of the discussion, leaving only 10–15% for control to govern. This picture is further amplified in the case of face-to-face contacts. Only the first 10% of discussion is dominated by orientation and control virtually never reaches dominance, gaining the upper hand by only a small margin in the final 10% of the talk.

It is fair to say that evaluation is the main focus of the face-to-face conversations. While the phases still follow their general trajectories (orientation—decay; evaluation—growth, maximum, decay; control—growth), the proportions are such that only evaluation gets clear focus. This is also present, alas to a smaller extent, in the phone condition. The more direct was the medium, the more time team members dedicated to exchange evaluations. This relatively high proportion of evaluation in voice media might be considered an indicator of a social process going on between team members. According to Bales [62], group dynamics reflects two essential types of needs of group members: the need to perform a task effectively (instrumental need) and the need to associate with others (socio-emotional or expressive need). We expect that in the media where the voice or the face of a partner is available, the socio-emotional need might be more pronounced than in chat. On the other hand, because of rehearsability and reprocessability of the messages when text-chatting (c.f. [63]) the meaning exchanged by the participants is more relevant and concise than in voice media [64,65].

We may conclude that the bandwidth of the medium not only determines the grand total percentages of each discussion phase, but also the time course of their development. The leanest medium forces users to curtail the evaluation phase (and their socializing needs) in order to maintain optimal task performance. As the medium’s bandwidth growths—from text to voice, from voice to face to face—the length of the domination of evaluation phase grows as well. The low cost of adding points to the negotiation process, of contributing one’s own evaluations enables the interlocutors to prolong this phase without losing on quality of solution and without increasing the time needed to reach it.

Our results add to the so far scarce literature on time patterns of interaction via various media. For example Poole and Holmes [66] have already shown that the type of group decision support system changes the structure of the decision path in teams communicating via those systems. Jonassen and Kwon [29] who compared text-based and face-to-face communication using Functional Category System by Poole and Holmes [66] have shown that conversations through computer conference had a more reiterative dynamics of problem solving. That is, communicators needed more repetitions of the sequence composed of problem definition—orientation—solution development, whilst face-to-face conversations tended to be more linear and included less problem solving activities. Another study [67] compared virtual and face-to-face teams by using Tuckman's model „forming, storming, norming, performing” [68]. The results have shown that the percent of time spent in each phase was similar in both conditions: both virtual and face-to-face teams spent similar proportions of time in each team formation stage.

It is worth noting that the bulk of the studies described above were carried out over 10 years ago (most importantly, the studies showing no differences between media in time patterns of communication). However, in the times of rapid technological advancements a 10-year long period renders most results on usage of technology at best outdated and sometimes even invalid. In light of the results presented here—where both the time to reach a solution as well as its quality were not dependent on the media but the time sequence of interaction phases was adjusted to media specifics—we conclude that contemporary ICT users are more apt at using the technology to their advantage. This difference from previously reported results may in part be due to different experimental settings but we can also hypothesize that the ubiquity and multi-functionality of new media, together with co-evolution of human behavior and technology have led to this increase in flexibility of communication behavior. However, to determine how much of this adjustment is due to long-term adaptation and how much due to cognitive flexibility, a longitudinal study would be required. Still, even in laboratory settings, we were able to observe that current technology users have no problems switching to whichever medium is available at a particular time and adjusting their communication practices to its particular strengths and weaknesses.

It would be interesting to follow with similar studies that would investigate this differential tuning in different contexts: interacting groups of various sizes and groups differing in the strength of interpersonal ties. One of the limitations of this study was that the group was defined in its minimal requirement—a dyad. It might occur that while dyadic interaction has become simple and intuitive through a variety of media, with larger groups some channels might still be preferred and might influence the group effectiveness and the quality of communication outcome. Another limitation comes from the fact that the group discussions in our study were task oriented and did not delve into other areas. This could be followed up by investigating if any medium is still preferred or is still most effective when different communication goals are at stake. For example, some group tasks might not require a convergent, optimal solution and rather might focus on the socializing aspect of communication or on opinion sharing and opinion formation.

Finally, another limitation comes from the fact that the age group range of the participants was limited—it would be interesting to compare the strategies of older age groups while communicating via different channels. It might occur that their behavior is not as flexible as of those ICT users that have grown up surrounded by technology. However, it is worth noting that the ICT mediated communication fluency in the younger group observed in this study is a phenomenon that can be expected to become more and more prevalent in general population both due to the spread of ICT usage within older age groups as well as due to generational change.

Our results put forward the need for higher focus of media researchers on the mezzo level of communication: not the micro level of single utterances or the macro level of the outcome but the level of conversation topics, team development phases or information processing activities. So far research in this area is acutely scarce; a typical research paradigm investigates how inputs, especially medium, influence outcomes—satisfaction and performance of communication partners. Our findings point out that this approach is losing its validity due to the increasing flexibility of users’ communication strategies in various media conditions.
